# Detection of *Opisthorchis viverrini* Infection among the ASEAN Population in Thailand Using a Verbal Screening Test and Fecal Concentrator Kit

**Published:** 2018

**Authors:** Natthawut KAEWPITOON, Soraya KAEWPITOON, Thirayu MEERERKSOM, Siwawich CHAN-ARAN, Wararat SANGWALEE, Jun NORKAEW, Jirayu CHUATANAM, Jirawoot KUJAPAN, Natnapa PADCHASUWAN, Taweesak TONGTAWEE, Likit MATRAKOOL, Ryan LOYD, Parichart WAKKHUWATTHAPONG

**Affiliations:** 1. Parasitic Disease Research Center, School of Medicine, Suranaree University of Technology, Nakhon Ratchasima, Thailand; 2. Suranaree University of Technology Hospital, Suranaree University of Technology, Nakhon Ratchasima, Thailand; 3. School of Family Medicine and Community Medicine, Institute of Medicine, Suranaree University of Technology, Nakhon Ratchasima, Thailand; 4. Dept. of Business Computer, Faculty of Management Science, Nakhon Ratchasima Rajabhat University, Nakhon Ratchasima, Thailand; 5. Dept. of Public Health, Faculty of Public Health, Vongchavalitkul University, Nakhon Ratchasima, Thailand; 6. Dept. of Public Health, Faculty of Liberal Arts and Science, Roi-Et Rajabhat University, Roi-Et, Thailand; 7. Dept. of Health Education, Faculty of Public Health, Khon Kaen University, Khon Kaen, Thailand

**Keywords:** *Opisthorchis viverrini*, ASEAN population, Thailand, Verbal screening test, Fecal concentrator kit

## Abstract

**Background:**

*Opisthorchis viverrini* is a serious health problem in Southeast Asia. The infection is associated with cholangiocarcinoma. Therefore, this study was aimed to detect *O. viverrini* infections among the ASEAN population in Thailand.

**Methods:**

A cross-sectional study was conducted among 249 individuals from ASEAN populations in Thailand including Thai, Laotian, Cambodian, and Myanmar. Participants were screened using the *O. viverrini* verbal screening test (OvVST). Fecal samples were processed by the mini-parasep sf parasite fecal concentrator.

**Results:**

The infection rate of *O. viverrini* was 27.21%. The majority of infections was detected in females, in the age group 31–40 yr old, in the primary school education level, and in the occupation of labor. By country, *O. viverrini* infection was detected more often in the Lao PDR (30.77%). In screening for *O. viverrini* infection, OvVST had a high sensitivity (93.48%), specificity (86.70%), NPV (98.32%), and accuracy (87.95%). The PPV was 61.43% for OvVTS. The observed agreement was substantial for OvVST (*k*-value = 0.64).

**Conclusion:**

*O. viverrini* infections are still detected in ASEAN countries therefore large scale active surveillance is required. OvVST had a high sensitivity, specificity, and accuracy for screening the risk groups for *O. viverrini*.

## Introduction

*Opisthorchis viverrini* remains a major public health problem in Southeast Asia particularly in Thailand, the Lao People’s Democratic Republic (PDR), Cambodia, and Vietnam (1). *O. viverrini* infection is associated with hepatobiliary diseases including hepatomegaly, cholangitis, cholecystitis, and gallstones (2–4). The infection is strongly correlated with cholangiocarcinoma (CCA), a bile duct cancer (5, 6).

Presently, the *O. viverrini* infection has been classified as Type 1 carcinogens by the International Agency for Research on Cancer, WHO (7). CCA is responsible for a major proportion of the burden of disease and death in Thailand, apart from causing hundreds of millions of people to surrender their rights to healthy and dignified lives (8). *O. viverrini-*induced CCA ranks first in mortality among cancers for men and second among cancers in women in the Mekong Basin sub-region (1, 8, 9).

In addition, *O. viverrini*-induced CCA is expected to increase sharply in the near future as a result of the demographic and economic factors occurring in Thailand, Lao PDR, Cambodia and Vietnam. The spread of liver fluke infection in the region due to increased migration among the ASEAN Economic Community (AEC) countries (Thailand, Laos, Cambodia, Vietnam and Myanmar) as a result of an open borders policy started in 2015 (8). For this reason, *O. viverrini* constitutes an important health problem in many parts of Southeast Asia and eradication of the fluke populations is urgently needed in these areas. Low cost and effective tool is needed for active surveillance among the risk group.

We aimed to screen the population at risk for *O. viverrini* and also detect the infection.

## Materials and Methods

A cross-sectional study was conducted among the total of 249 participants including Thai, Cambodian, Laotian, and Burmese, who work or habitat in Nakhon Ratchasima province, in northeastern region of Thailand during August 2016 to February 2017, were enrolled.

All participants provided informed consent before participating in the study. This study was performed in accordance with good clinical practice and the guidelines of the Declaration of Helsinki. Ethical clearance was obtained from the Ethics Committee for Research Involving Human Subjects, Suranaree University of Technology (EC-59–39).

**Fig. 1:**
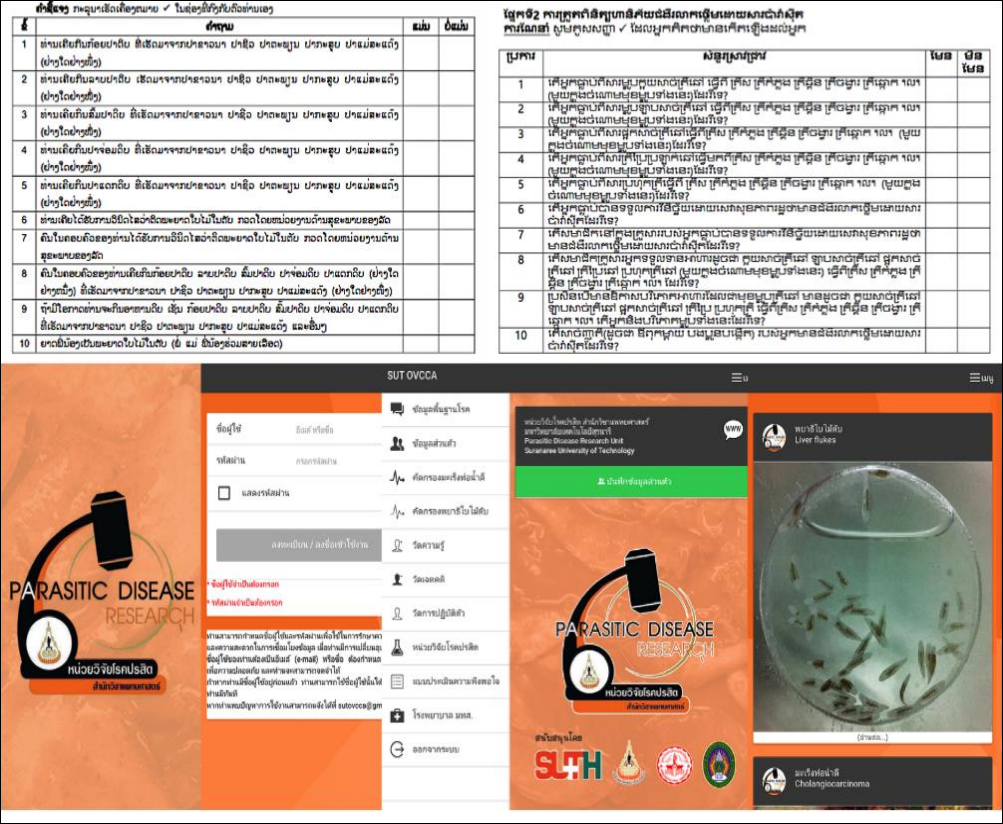
The *Opisthorchis viverrini* verbal screening test; OvVST, was used for screening the population at risk for *O. viverrini* infection

Populations at risk for *O. viverrini* infection were screened by the mini-verbal screening questionnaires; Ov verbal screening test: OvVST, through interviewed or self-checked using paper or mobile application. OvVST was created and literature reviewed from the basic knowledge that related to life cycle of *O. viverrini*, and then translated to Laotian and Cambodian language. For Burmese, translator was needed to translate for participants. Meanwhile, the participant could be self-checked by themselves through mobile application; SUT-OVCCA application in iOS and android platform (Fig. 1). OvVST is contained 1) the general information including gender, age, education, marital status, and nationality, and 2) the question with yes/no choices related to history of (i) consumption of raw spicy salad cyprinoid fish, (ii) consumption of raw minced cyprinoid fish, (iii) consumption of raw prickled cyprinoid fish, (iv) consumption of raw preserved small cyprinoid fish, (v) consumption of raw fermented cyprinoid fish, (vi) diagnosed as the opisthorchiasis, (vii) family member had diagnosed as the opisthorchiasis, (viii) family member consumed the various dishes of raw cyprinoid fish, (ix) trend to consume the various cyprinoid fish, and (x) relative family had diagnosed a cholangiocarcinoma. The OvVST was tested and tryout before the study, and then analyzed for the reliability (Cronbach alpha coefficient was 0.75).

Fecal specimens were collected, processed and examined following the manufacturer instructions for the mini-parasep sf fecal parasite concentrator, a new fecal parasite concentrator developed by the company DiaSys Europe Limited (formerly Intersep Ltd). The tubes and the sedimentation cones were labeled with the specimen identification numbers. A level fecal sample was introduced into each tube containing 3.3 ml of 10% formol-saline using the spoon on the end of the Mini Parasep SF filter. The MPFC was sealed by screwing in the filter/sedimentation cone unit. This was then vortexed for emulsification with the sedimentation cone pointing upwards. The MPFC was then inverted and centrifuged at 1,500 rpm for 2 minutes. The mixing chamber and the filter were then unscrewed and discarded for incineration while the supernatant in the sedimentation cone was decanted. The deposit was then examined microscopically using physiological saline and iodine for the eggs and larvae of intestinal parasites. Each of the preparations was examined systematically under the microscope for a minimum of 5 minutes. All preparations were initially screened with a low-power (10×) objective lens. Suspected parasitic objects were subsequently examined under a high-power (40×) objective.

Stool samples were examined by two laboratory technologists and then confirmed by an expert parasitologist. Finally, the data were analyzed and interpreted accordingly. Patients who were infected with other known parasites were treated with anti-parasitic drugs and also attended health education.

Data entry and analysis were done using Excel and SPSS version 22.0 (Chicago, IL, USA). The risk score was calculated following 1+2+3+4+5+6+7+8+9+10, and then interpreted as high risk (8–10 points), moderate risk (4–7 points), low risk (1–3 points), or no risk (0 point). Infection rate, sensitivity, specificity, positive and negative predictive values, and accuracy were analyzed by the SPSS and kappa estimator was employed to determine the strength of the agreement of each methods with the combined result. Kappa values were interpreted as follows: 0.01–0.20 slight agreement, 0.21–0.40 fair agreement, 0.41–0.60 moderate agreement, 0.61–0.80 substantial agreement, and 0.81–0.99 perfect agreement (10).

## Results

Two hundred forty-nine participants were enrolled. The majority of participants were male (57. 30%), aged 21–30 yr old (34.54%), highest education primary school (76.71%), married (72.29%), and laborers (75.90%). Cambodians formed the largest ethnic group (48.59%). The *O. viverrini* infection rate was 18.47%. The majority of *O. viverrini* infections were found in females (19.63%), aged 31–40 yr old (36.00%), uneducated (26.92%), laborers (21.69%), and were divorced (26.67%). By nationality, *O. viverrini* infections were detected in Laos (30.77%), Cambodia (25.62%), and Thailand (5.56%) (Table 1).

**Table 1:** General characteristics of 249 ASEAN populations in Nakhon Ratchasima province, Northeast, Thailand

***General characteristics***	***No.***	***O. viverrini***	**%**
Gender			
Male	142	25	17.61
Female	107	21	19.63
Age (yr)			
15–20	26	4	15.38
21–30	86	11	12.79
31–40	50	18	36.00
41–50	38	9	23.68
51–60	20	1	5.00
>60	29	3	10.34
Education			
Uneducated	26	7	26.92
Primary School	191	36	18.85
Secondary School	28	3	10.71
Undergraduate	4	0	0.00
Occupation			
Labor	189	41	21.69
Agriculture	45	2	4.44
House Keeper	10	2	20.00
Other	5	1	20.00
Marital Status			
Single	54	3	5.56
Married	180	39	21.67
Divorced	15	4	26.67
Nationality			
Thailand	54	3	5.56
Cambodia	121	31	25.62
Laos	39	12	30.77
Myanmar	35	0	0.00
**Total**	249	46	18.47

The helminthic eggs found in the fecal samples from the 249 participants, as well as their respective frequencies, are shown in Table 2, according to the diagnostic method used. From the total enrolled study participants 23.69% (59/249) were infected by one or more helminthic infection. By species the detected parasites were *O. viverrini* 18.47%, followed by *Endolimax nana* (1.61%), and hook-worm (1. 16%).

**Table 2:** Frequency of helminthic eggs and larvae detected by the mini parasep sf parasite fecal concentrator in fecal samples from 249 ASEAN population participants in Nakhon Ratchasima, Northeast, Thailand

***Parasitic infections***	***No. of infection***	***%***
*Opisthorhis viverrini*	46	18.47
*Endolimax nana*	4	1.61
Hookworm	4	1.61
*Strongyloides stercolaris*	3	1.20
*Taenia* spp.	3	1.20
*Blastocystis hominis*	1	0.40
*Entamoeba histolytica*	1	0.40
**Total**	**59**	**23.69**

The populations at risk for *O. viverrini* infection were classified into varying risk and no risk groups; the largest group had moderate risk (38.57%), followed by low risk (32.86%), and high risk groups (28.56%). The stool samples of the populations at risk for *O. viverrini* infection were examined and we found 43 positive cases and 27 negative cases. Positive cases were classified as moderate (20 cases), high risk (18 cases), and low risk (5 cases). Meanwhile, the no risk group of 179 participants was tested and only 3 were found to have *O. viverrini* on examination (Table 3).

**Table 3:** Populations at risk for *Opisthorchis viverrini* infection among 249 ASEAN populations participants in Nakhon Ratchasima of Thailand, were screened using the *O. viverrini* verbal screening test

***Risk Group***	***No. of risk or no risk***	***O. viverrini infection***			
		**No. of positive**	**No. of negative**
Risk	70	43	27
High Risk	20	18	2
Moderate Risk	27	20	7
Low Risk	23	5	18
No Risk	179	3	176
**Total**	**249**	**46**	**203**

Fecal diagnosis results based on the fecal concentrator kit were used as the gold standard to estimate the sensitivity, specificity, NPV and PPV, and accuracy of the OvVST methods for screening the population at risk for *O. viverrini* infection (Table 4). The parameters measured for OvVST were as follows; sensitivity (93.48%), specificity (86.70%), PPV (61.43%), NPV (98.32%), and accuracy (87.95%). The agreement of OvVST with the comparison between screening results and fecal detecting results was calculated by the MPFC method. The observed agreement was substantial for OvVST (k-value = 0.64, mean rank =0.51–0.74).

**Table 4:** Sensitivity, specificity, negative predictive value, positive predictive value, and accuracy of the *O. viverrini* verbal screening test among 249 ASEAN populations in Nakhon Ratchasima, Northeast, Thailand

***Parameters***	***%***	***95% CI***
Sensitivity	93.48	89.37–96.42
Specificity	86.70	71.93–92.75
Negative predictive value	98.32	90.32–99.86
Positive predictive value	61.43	51.45–71.14
Accuracy	87.95	81.22–93.14

## Discussion

ASEAN was founded in 1967 in order to promote economic and cultural development, to promote trade, industrial, agricultural, and scientific collaboration, and to promote peace and stability in the region (11). Ten member states make up ASEAN including Brunei Darussalam, Cambodia, Indonesia, the Lao PDR, Malaysia, Myanmar, the Philippines, Singapore, Thailand, and Viet Nam. The ASEAN Economic Community countries have had an open borders policy since 2015. These countries also harbor a mostly hidden burden of poverty and neglected tropical diseases (NTDs).

The three major intestinal helminth infections are the most common NTDs; each type of helminthiasis is associated with approximately 100 million infections in the region. In addition, more than 10 million people suffer from either liver or intestinal fluke infections (12). Pullan et al. (13) in reporting on soil-transmitted helminth infections determined that 126.7 million people in Southeast Asia are infected with Ascaris roundworms, while 115.3 million are infected with Trichuris whipworms, and 77.0 million have hookworm infections (14,15). Thus, approximately one-half of the people of Southeast Asia living in poverty have one or more soil-transmitted helminth infections.

In this study, 23.69% were infected by one or more helminthic infection. The majority of infections were *O. viverrini*, followed by hook-worm, *Endolimax nana, Strongyloides stercoralis, Taenia* spp*., Blastocystis hominis,* and *Entamoeba histolytica*. This study shows a higher prevalence than a previous study. In Myanmar migrant workers in Thailand the overall prevalence of intestinal parasitic infections was 13.6%. The migrant workers were mainly infected with the fecal-oral transmitted parasites; *E. histolytica / dispa* (3.8%), *A. lumbricoides* (3.3%), and *T. trichiura* (2.3%) (16). The total infection rate was low among this group when compared to other studies. Sagnuankiat et al. (17) in reporting on the prevalence of intestinal parasitic infections among 372 immigrant children at 8 child-daycare centers during their parents’ work time, by fecal examination found intestinal parasitic infections highly prevalent, at 71.0%. These infections comprised both helminths and protozoa: *Trichuris trichiura* (50.8%), *Enterobius vermicularis* (25.2 %), *A. lumbricoides* (15.3 %), hookworm (11.6 %), *Giardia lamblia* (10.2 %), *E. nana* (3.5 %), *E. coli* (2.7 %), and *B. hominis* (0.5%). Among the liver fluke infections, Furst et al. (18) estimated that 9.3 million people suffer from bile duct liver fluke infections in the region (39% of the global number of cases), including 8.03 million cases of opisthorchiasis, mostly in the Lao PDR and Thailand. This study is the first report on *O. viverrini* infection rates among ASEAN populations in Thailand.

The results indicate that migrant workers, particularly Laotian and Cambodian, should be large scale screened to prevent and control *O. viverrini* transmission. *O. viverrini* is a common parasite found in central and southern Laos and constitutes a major public health problem in the country. The Lao people continue to have the habit of extensively consuming raw or half-cooked fish, which are intermediate hosts. In Khammouane Province, the infection rate with *O. viverrini* was 54.8%. Factors associated with *O. viverrini* infections were gender, a habit of defecation in fields, and raw fish consumption (19). The overall liver/intestinal helminth egg positive rate was 71.9% among 6,178 residents in 9 provinces, Lao PDR. *O. viverrini*/minute intestinal fluke revealed the highest prevalence (55.6%); the endemic regions were Savannakhet, Khammouane, Vientiane (Nam Ngum), Champasak (Khong Island), and Saravane Province (20). The results of this study highlight that *O. viverrini* is of current public health significance in different areas of the Lao PDR. Meanwhile, investigation of the status of intestinal helminthic infections in Cambodia has been carried out on a national scale, including 19 provinces. The overall egg positive rate of intestinal helminths was 26.2% among 32,201 schoolchildren and adults. The prevalence of *O. viverrini*/minute intestinal fluke was 5.7%. The central and southern areas, in particular Takeo and Kampong Cham Provinces, showed a high prevalence of *O. viverrini*/minute intestinal fluke (23.8–24.0%) (21). Surveillance was also conducted among 16,082 fecal samples in 55 villages in five Cambodian provinces. Of these 1232 were egg positive. In the 15 villages, having egg-positive rates of greater than 10%, eggs were found in 998 of 3585 stool samples, for an egg-positive rate of 27.8% (22). In Thailand, a national survey was performed for *O. viverrini* infection and found an overall prevalence of helminthiasis among 15,555 Thai people of 18.1%. The highest prevalence was found in the northeastern regions of Thailand. The majority of detected parasites were *O. viverrini* (1,351 cases, 8.7%) (23). Meanwhile, in Nakhon Ratchasima province of Thailand the reported rates of *O. viverrini* infection in various studies were 2.01% (24), 2.82% (25), and 2.48% (26). The present study also indicates that *O. viverrini* is still a problem in Thailand, the Lao PDR, and Cambodia, but is not a serious problem in Myanmar.

The countries that comprise ASEAN have experienced impressive economic growth in recent years. However, such rapid growth has also left a substantial fraction of people economically marginalized. Overall, almost 200 million people in ASEAN countries, or roughly 30% of the population, live in extreme poverty, i.e., on less than US$2 per day, or below their national poverty lines (27–29). Countries also harbor a mostly hidden burden of neglected tropical diseases (NTDs). Of the almost 200 million people who live in extreme poverty in ASEAN countries, mostly in the low or lower middle-income countries of Indonesia, the Philippines, Myanmar, Viet Nam, and Cambodia, many of them are affected by at least one NTD. For this reason, OvVST was developed and created for iOS and android platforms, as well as paper questionnaires. Fecal diagnosis results based on the fecal concentrator kit were used as the gold standard. The observed agreement between screening results and fecal detecting results by the MPFC method was substantial for the OvVST (k-value = 0.64, mean rank =0.51–0.74). OvVST is able to identify the populations at risk for *O. viverrini* infection, as it successfully detected 43 positive cases but only had 3 false negative cases. Of 27 participants who had scores in the risk group but were negative for *O. viverrini* infection, they possibly had treatment before participating in the screening project or they consumed raw fish that did not contain the infective stage of the parasites. This indicates that using OvVST for screening for *O. viverrini* infection, is both very simple to answer and very fast to analyze by themselves. Participants took only about 2 minutes/person to answer the questions and they were then able to calculate their own risk using the test.

The impact of intestinal parasitic infections on public health is well known; they can spread from infected immigrant areas to uninfected areas via close contact and fecal-oral transmission from contaminated food and water. These results indicate that intestinal helminth infections are a serious public health problem.

## Conclusion

OvVST is a simple and fast screening test with low cost. This tool may useful for *O. viverrini* screening for the large scale prevention and control of the spread of this liver fluke.
